# NK- and T-cell granzyme B and K expression correlates with age, CMV infection and influenza vaccine-induced antibody titres in older adults

**DOI:** 10.3389/fragi.2022.1098200

**Published:** 2023-01-05

**Authors:** Chris P. Verschoor, Emilie Picard, Melissa K. Andrew, Laura Haynes, Mark Loeb, Graham Pawelec, George A. Kuchel

**Affiliations:** ^1^ Health Sciences North Research Institute, Sudbury, ON, Canada; ^2^ Northern Ontario School of Medicine, Sudbury, ON, Canada; ^3^ Department of Medicine, Dalhousie University, Halifax, NS, Canada; ^4^ UConn Center on Aging, University of Connecticut School of Medicine, Farmington, CT, United States; ^5^ Department of Pathology and Molecular Medicine, McMaster University, Hamilton, ON, Canada; ^6^ Department of Immunology, University of Tübingen, Tübingen, Germany

**Keywords:** aging, granzyme B, granzyme K, NK-cells, t-cells, CMV, influenza, vaccination

## Abstract

Granzymes are a family of serine-proteases that act as critical mediators in the cytolytic and immunomodulatory activities of immune cells such as CD8^+^ T-cells and natural killer (NK) cells. Previous work indicates that both granzyme B (GZB) and K (GZK) are increased with age in CD8^+^ T-cells, and in the case of GZB, contribute to dysfunctional immune processes observed in older adults. Here, we sought to determine how GZB and GZK expression in NK-cells, and CD4^+^, CD8^+^, and gamma-delta T-cells, quantified in terms of positive cell frequency and mean fluorescence intensity (MFI), differed with age, age-related health-traits and the antibody response to high-dose influenza vaccine. We found that the frequency and MFI of GZB-expressing NK-cells, and CD8^+^ and Vδ1+ T-cells, and GZK-expressing CD8^+^ T-cells was significantly higher in older (66–97 years old; *n* = 75) vs. younger (24–37 years old; *n* = 10) adults by up to 5-fold. There were no significant associations of GZB/GZK expression with sex, frailty or plasma levels of TNF or IL-6 in older adults, but those who were seropositive for cytomegalovirus (CMV) exhibited significantly higher frequencies of GZB+ NK-cells, and CD4^+^, CD8^+^ and Vδ1+ T-cells, and GZK+ CD8^+^ T-cells (Cohen’s d = .5–1.5). Pre-vaccination frequencies of GZB+ NK-cells were positively correlated with vaccine antibody responses against A/H3N2 (d = .17), while the frequencies of GZK+ NK and CD8^+^ T-cells were inversely associated with A/H1N1 (d = −0.18 to −0.20). Interestingly, GZK+ NK-cell frequency was inversely correlated with pre-vaccination A/H1N1 antibody titres, as well as those measured over the previous 4 years, further supporting a role for this subset in influencing vaccine antibody-responses. These findings further our understanding of how granzyme expression in different lymphoid cell-types may change with age, while suggesting that they influence vaccine responsiveness in older adults.

## 1 Introduction

The phenotypic and functional landscape of immunity changes in numerous ways as we age. Although often described as “immunosenescence”, a term that echoes connotations of disability and diminished capacity, these alterations are much more nuanced and therefore are better framed as a context- and individual-specific dysregulation of multiple cellular and molecular processes. One of these critical processes is the cytolytic response of T-cells and natural killer (NK) cells. They are chiefly responsible for the clearance of host-cells that express markers of “non-self”, and as such, play important roles in preventing and reducing tumour burden and resolving viral infections. In the case of T-cells, namely those of the cytotoxic CD8 lineage, this mainly occurs in an MHC-restricted, antigen-specific manner *via* recognition through the T-cell receptor, while NK-cells rely on interactions with activating and inhibitory receptors on the host cell surface. Activation in this manner commonly results in the release of cytotoxic granules containing the pore-forming glycoprotein perforin, which facilitates entry into the target cell, and a family of serine-proteases collectively known as granzymes, which act to modulate intracellular function, most often leading to cell death (Voskoboinik et al., 2015).

Five human granzymes have been identified, A, B, H, K and M, the most abundant and well-studied being A and B (Hendel et al., 2010). Granzyme A and B (GZB) have been shown to be present in increased amounts with increasing age in human platelets (Campbell et al., 2018) and CD8^+^ T-cells (Mellor-Heineke et al., 2013; Zöphel et al., 2022), respectively, and recently a population of CD8^+^ T-cells expressing granzyme K (GZK) was shown to be increased in number across multiple tissues in older mice and in human blood (Mogilenko et al., 2021), particularly the central memory and CD57^+^ effector memory subsets (Mogilenko et al., 2021). Although the consequences of increased GZK with age are unknown, it has been implicated as a pathogenic factor in burn injury (Turner et al., 2019), tuberculosis (Cai et al., 2022), and multiple sclerosis (Rodríguez-Lorenzo et al., 2022). Granzyme B has been implicated in the incidence and severity of a number of pathological conditions, including viral and bacterial infection (García-Laorden et al., 2016; Adam et al., 2021; Verschoor et al., 2021) and age-related dysfunctions in wound healing (Turner et al., 2021).

It is unclear why GZB and GZK expression in CD8^+^ T-cells differs with age and whether these observations can be extended to other cell types that commonly express them, such as NK-cells. Hence, in the following study we aimed to compare the expression of GZB and GZK in NK-cells, and CD4^+^, CD8^+^ and gamma-delta (γδ) T-cells between young and older adults and assess its association with age-related factors such as frailty, CMV serostatus and chronic inflammation. Considering the multifaceted role of granzymes as immunomodulators (Omoto et al., 2010; Wensink et al., 2014; Shimizu et al., 2019; Kaiserman et al., 2022), we also sought to investigate the association of cell-specific GZB/GZK expression with influenza vaccine-induced antibody production in older adults.

## 2 Materials and methods

### 2.1 Participants and sample collection

In late 2018, adults aged <40 (*n* = 10) or >65 years (n = 75) were recruited at the Health Sciences North Research Institute (HSNRI) from the communities of Greater Sudbury, Ontario, Canada, and at UConn Health through the University of Connecticut Center on Aging Recruitment Core from the communities belonging to and surrounding Hartford, Connecticut, United States. Participants needed to be vaccinated in the previous influenza season to participate and were excluded if they had a known immunosuppressive disorder or were taking immunosuppressive medications including prednisone in doses >10 mg/day, or had a previous severe reaction to the vaccine due to egg, latex, or thimerosol allergies. Following informed consent, study participants were characterized according to demographics, body-mass index (BMI), chronic medical conditions, and functional impairments. A frailty index (FI) was calculated based on 40 items representing accumulated health deficits across multiple systems (Loeb et al., 2020). The FI is a ratio that counts the proportion of health deficits an individual has relative to the total number considered (i.e., 40), and generally ranges between 0 and .7 (Verschoor and Tamim, 2019).

The study protocol was approved by the Institutional Review Board of the University of Connecticut Health Centre (UCHC) and the Health Sciences North Research Ethics Board and all study participants provided written informed consent to participate in the study.

### 2.2 Sample collection, influenza vaccination and antibody titre measures

All participants provided blood in heparinized and coagulant-free vacutainers following consent; older adults also provided blood 4-, 10- and 20-week later. From this, peripheral blood mononuclear cells (PBMCs) were isolated using Ficoll-Plaque Plus (GE Healthcare, IL, United States) gradient purification, resuspended in 90% human AB serum (Corning, NY, United States) and 10% DMSO, and transferred to liquid nitrogen for long-term storage. Plasma and serum were also isolated and stored at −80°C.

Older adults received the Fluzone (Sanofi Pasteur) high-dose trivalent vaccine at the time of consent. This formulation contained antigens from the following strains: A/HongKong/4801/2014, A/Michigan/45/2015 and B/Colorado/06/2017. Antibody titres were measured in serum by hemagglutination inhibition (HAI) assay using previously-described standard methods (WHO Manual on Animal Influenza Diagnosis and Surveillance, 2002; Loeb et al., 2020).

### 2.3 Serological measures and flow cytometry analysis

TNF and IL-6 were quantified in participant plasma pre-vaccination using the Ella Automated Immunoassay System and Simple Plex 2nd generation Human TNF and IL-6 cartridges (R&D Systems, MN, United States); this has been previously described (Picard et al., 2022). CMV serostatus was determined in serum using a CMV IgG ELISA kit (Genesis Diagnostics Ltd., Littleport, United Kingdom) according to the manufacturer’s instructions.

**FIGURE 1:**
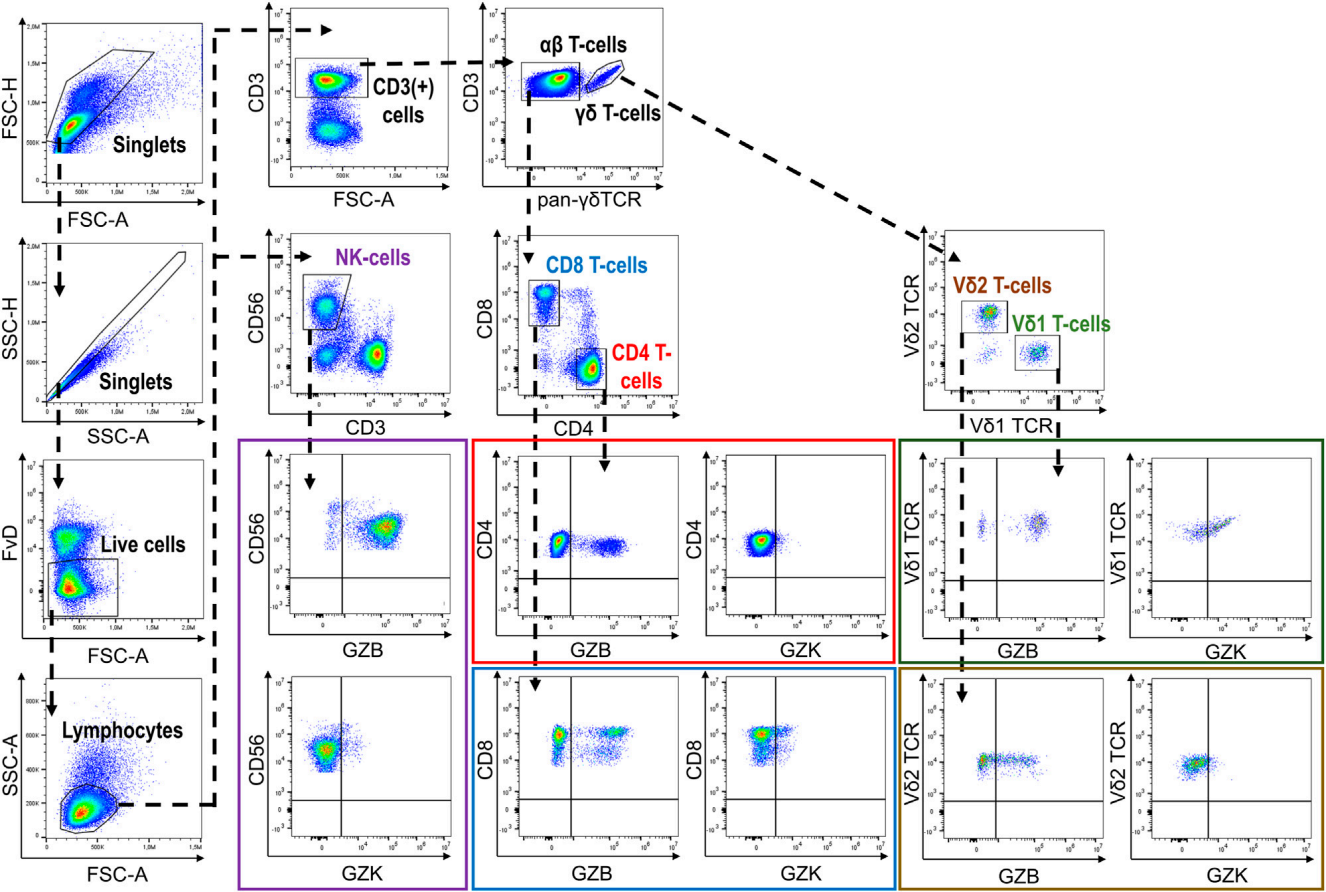
Gating strategy depicting the identification of GZB- and GZK-expressing NK-cells and CD4^+^, CD8^+^ and γδ^+^T-cells.

Following thawing, PBMCs were resuspended in flow cytometry staining buffer (PBS, 2% FBS, 2 mM EDTA, .01% NaN3) and incubated first with Fixable Viability Dye (FvD)-eFluor780 (eBioscience, CA, United States) and 10% heat-inactivated human AB serum (Sigma-Aldrich, MO, United States) for 15 min at 4°C. After washing with flow cytometry staining buffer, PBMCs were stained with purified-γδ-TCR (BD Biosciences, NJ, United States) and subsequently with F(ab’)2-goat anti-mouse IgG-APC (eBioscience, CA, United States) for 20 min at 4°C. Following incubation in flow cytometry staining buffer containing 10% mouse serum (R&D Systems, MN, United States) for 10 min at 4°C, PBMCs were washed with flow cytometry staining buffer then incubated with CD3-AF700, CD4-BV650, CD8-PerCp-Cy5.5, and CD56-PE-Dazzle (Biolegend, CA, United States) antibodies for 20 min at 4°C. PBMCs were then incubated with Fixation/Permeabilization solution (BD Biosciences, NJ, United States) for 20 min at 4°C, followed by washing and incubation with Granzyme B-FITC, Granzyme K-PerCpCy5.5 and Perforin-PE-Cy7 (Biolegend, CA, United States) antibodies for 30 min at 4°C. PBMCs were then resuspended in Perm/Wash buffer (BD Biosciences, NJ, United States) and immediately analyzed on a CytoFLEX flow cytometer (Beckman Coulter, CA, United States). Analysis of resultant data was performed using FlowJo v.10.1.5. Initially, duplicates were removed *via* progressive gating on FSC-area vs. FSC-height and SSC-area vs. SSC-height. Next, dead cells were excluded by considering only FvD-negative cells and then a morphological gate (FSC-area vs. SSC-area) was used to identify the lymphocyte population. Major cell subsets and expression of GZB and GZK were subsequently quantified according to our gating strategy (Figure 1). GZB and GZK expression is given as the frequency of each respective subset that was positive for each of or both markers, and also as the mean fluorescence intensity (MFI) for the positive fraction of cells. Data points found to be more than 4 standard deviations from the cohort mean (a total of *n* = 9 data points across 6 measures) and were disproportionately influential on regression estimates in preliminary analyses [i.e., Cook’s distance >1 (Stevens, 1984)], were subsequently removed prior to the final analysis.

### 2.4 Retrospective analysis of the standard-versus high-dose vaccine randomized trial

All but one older adult in the current 2018/19 cohort originally participated in at least 1 year of a randomized trial to compare the Fluzone quadrivalent standard-dose to trivalent high-dose seasonal influenza vaccine (ClinicalTrials.gov #NCT02297542), conducted between 2014 and 2018 and previously described elsewhere (Loeb et al., 2020); *n* = 12, 5, 9 and 48 participants from the current cohort took part in 1, 2, 3 or all 4 years of that trial, respectively. The protocol for that trial matched that of the 2018/19 cohort, except for the double-blind randomization. Hence, blood was collected prior to vaccination and at 4-, 10- and 20-week post-vaccination, and HAI antibody titres were measured for the following strains: Year 1 (2014/15), A/Texas/50/2012, A/California/7/2009 and B/Massachusetts/2/2012; Year 2 (2015/16), A/Switzerland/9715292-2013, A/California/7/2009 and B/Phuket/3073/2013; Year 3 (2016/17), A/Hong Kong/4801-2014, A/California/7/2009 NYMC X-179A and B/Brisbane/60/2008; and Year 4 (2017/2018), A/HongKong/4801/2014, A/Michigan/45/2015 and B/Brisbane/60/2008.

### 2.5 Statistical analysis

Our analyses can be broadly grouped into investigating two objectives: first, to compare cell frequencies and granzyme expression between young and older adults, and second, to estimate associations of these measures with participants factors, vaccine responses, and absolute antibody titres at different time points. Continuous data were summarized as the median and interquartile range or geometric mean and standard deviation, and categorical values as the count and frequency. Comparisons between age groups were performed by Kruskal-Wallis or Fisher’s exact tests (i.e., participant factors) or Student’s t-test on log-transformed continuous variables (i.e., cell frequencies). Associations between GZB and/or GZK expression and participant factors or vaccine responses were estimated using linear regression and expressed in terms of Cohen’s d (Lakens, 2013). For age, sex, FI, CMV serostatus, TNF and IL-6 in older adults, the standardized (where mean = 0 and standard deviation = 1), log-transformed GZB/GZK value was regressed on a single factor while adjusting for participant site. All continuous factors other than age were also expressed as standardized, log-transformed values. For this set of analyses, *p*-values were corrected to maintain a false discovery rate (FDR) of 5% using the Benjamini-Hochberg procedure, grouped according to the participant factor (i.e., 5 cell subsets across 2 granzyme measures). For vaccine antibody responses, the standardized, log-transformed 4-week titre was regressed on the standardized, log-transformed GZB/GZK value, adjusting for the log-transformed baseline titre, age, sex and site; for titres at each time point, log titres were modeled as above, without adjusting for the baseline titre. For these analyses, multiple testing correction was performed on *p*-values grouped by viral (sub)type.

To estimate associations between the frequency of GZK+ NK- or CD8^+^ T-cells measured in the current 2018/19 cohort and retrospective A/H1N1 antibody titres obtained in the SD vs. HD randomized trial, we performed linear regression within separate years and on all years combined. Within each year, the standardized log antibody titre at a given time point was regressed on the standardized log GZK frequency, adjusting for age, sex, site and vaccine dose. For associations across years, linear mixed models were fitted using the ‘lme4’ package in R, including the fixed effects of age, sex and dose, and random intercepts for site, year and participant, with Nelder-Mead optimization. For these analyses, multiple testing correction was grouped according to year or model.

All analyses were performed in R v4.0.

## 3 Results

### 3.1 Age-related differences in cell subset frequency and granzyme B and K expression

**FIGURE 2:**
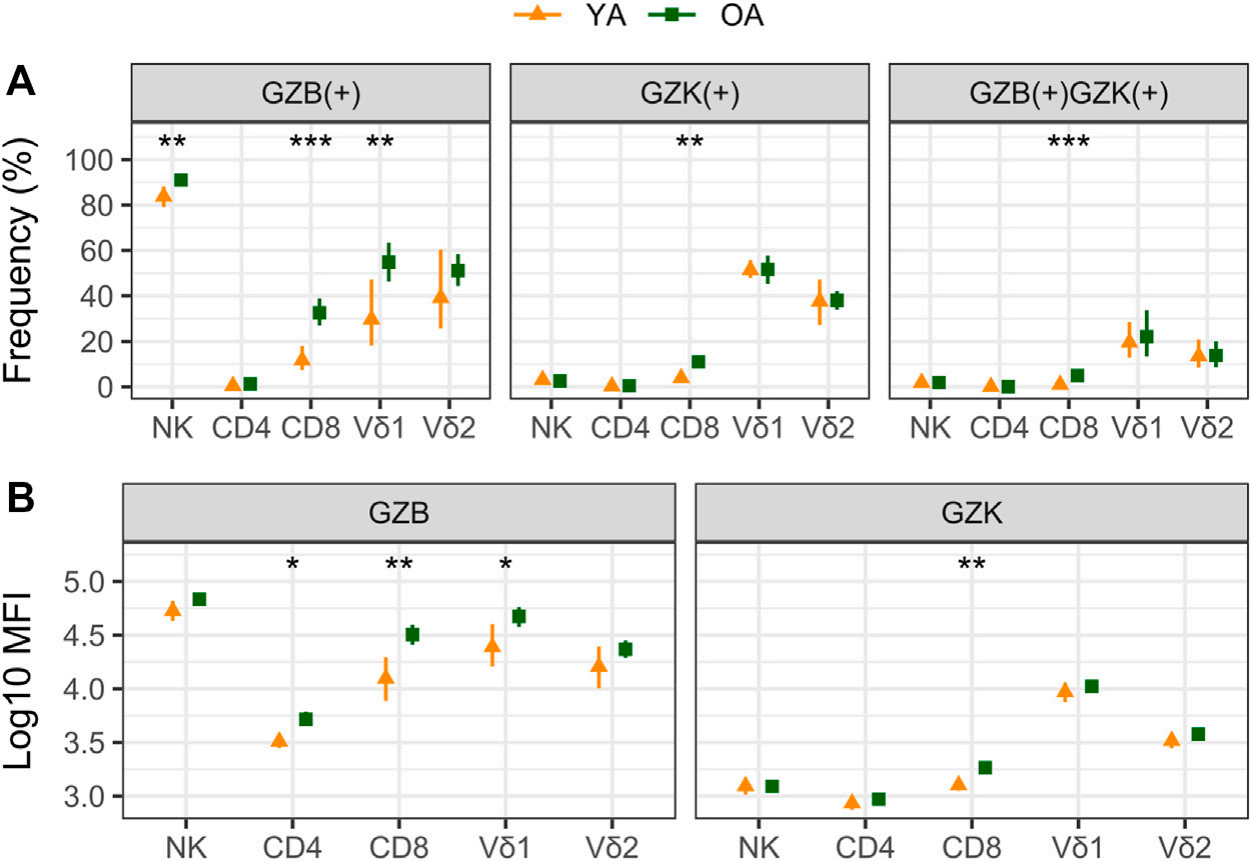
A comparison of granzyme B (GZB) and K (GZK) expression in young (YA) and older (OA) adults. The geometric mean and 95% confidence interval for **(A)** the frequency of GZB, GZK or GZB/GZK-expressing cells, and **(B)** the mean fluorescence intensity (MFI) of GZB or GZK expression within those populations are shown, with the significance of age-related differences determined by *t*-test on log-transformed values. ***, *p* < .001; **, *p* < .01; *, *p* < .05.

Our cohort included 10 young adults aged 24–37 and 75 older adults aged 66–97 years (Table 1). Plasma levels of TNF and IL-6 were significantly higher in older adults and the median and interquartile range for the frailty index in older adults was .08 (.032–.128), which can be considered relatively low. Of the cell subsets measured (Figure 1), the frequencies of CD8^+^ and Vδ2+ γδ T-cells were found to be significantly lower in older adults, while NK-cells were significantly higher (Table 2). Regarding the frequency of those subsets expressing either GZB or GZK, GZB+ NK- and Vδ1+ T-cells were significantly higher in older adults (young vs. older, geometric mean [95% CI]: NK = 84% [79, 89] vs. 91% [89, 93], Vδ1 = 30% [17, 53] vs. 55% [47, 65]), and CD8^+^ T-cells expressing GZB (12% [7, 20] vs. 33% [27, 39]), GZK (4% [2, 7] vs. 11% [9, 14]) or both granzymes (1% [.6, 1.5] vs. 5% [3.8, 6.5]) were significantly higher in older adults (Figure 2A; Supplementary Table S1). Age-related differences in the amounts of both granzymes (i.e., mean fluorescence intensity, MFI) mostly paralleled the frequency of positive cells, with the exception of the GZB MFI of CD4^+^ T-cells, which was also significantly greater in older adults (Figure 2B; Supplementary Table S1).

**TABLE 1:** Summary of demographics and health-related traits in young and older participants.

	Young adults	Older adults	
	(N = 10)	(N = 75)	*p*
Age	32 (27–35.5)	76 (71.5–83.5)	<0.001
Sex			1.000
Female	6 (60.0%)	48 (64.0%)	
Male	4 (40.0%)	27 (36.0%)	
Site			1.000
HSNRI	5 (50.0%)	37 (49.3%)	
UCHC	5 (50.0%)	38 (50.7%)	
Body-mass index	28 (24.6–30.7)	27 (24.4–31)	0.692
Frailty index	—	0.08 (0.032–0.128)	—
CMV serostatus			0.737
Negative	5 (50.0%)	31 (41.3%)	
Positive	5 (50.0%)	44 (58.7%)	
TNF (pg/ml)	8 (6.45–9.14)	11 (8.8–12.8)	<0.001
IL-6 (pg/ml)	1 (0.819–1.85)	3 (1.78–3.79)	0.012

Continuous data presented as the median and interquartile range, and categorical data as count and frequency. Differences between age groups tested by Kruskal-Wallis or Fisher’s exact test, respectively. Note, the frailty index was not measured in younger adults.

**TABLE 2:** The geometric mean and standard deviations of each cell type as the percentage relative to total lymphocytes.

	Young adults (N = 10)	Older adults (N = 75)	*p*-value
CD4^+^ T-cells	46.8 (1.15)	45.3 (1.26)	0.675
Missing	0 (0%)	13 (17.3%)	
CD8^+^ T-cells	22.4 (1.18)	16.3 (1.59)	0.037
Missing	0 (0%)	13 (17.3%)	
Vδ1+ T-cells	0.4 (1.77)	0.238 (3.17)	0.171
Missing	0 (0%)	13 (17.3%)	
Vδ2+ T-cells	1.08 (2.62)	0.274 (4.54)	0.007
Missing	0 (0%)	13 (17.3%)	
NK-cells	8.24 (1.54)	13.1 (1.67)	0.009
Missing	0 (0%)	12 (16.0%)	

Comparison of cell frequencies between younger and older adults performed by *t*-test on log-transformed values.

### 3.2 Factors associated with granzyme B and K expression in older adults

**FIGURE 3:**
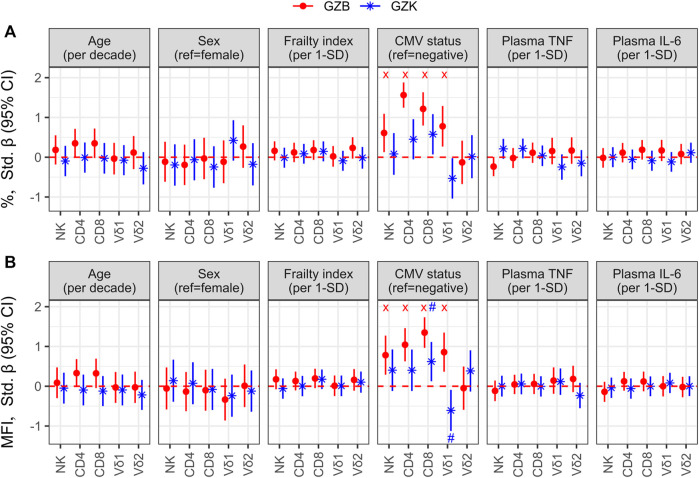
Factors associated with GZB and GZK expression in older adults. **(A)** The log frequency of GZB or GZK-expressing cells and **(B)** the log mean fluorescence intensity (MFI) of each population was regressed on age, sex, frailty index, CMV serostatus, and plasma TNF and IL-6, while adjusting for site of enrollment. For sex and CMV status, contrasts are males vs. females and positive vs. negative participants, respectively. For all others, coefficients are relative to the magnitude of increase shown in brackets. No overlap of the 95% CI with the red dotted line indicates that the association is significant at the nominal level, while the ‘x’ and ‘#’ indicate that the respective associations for GZB or GZK maintained significance at an FDR adjusted *p* < .05.

To better understand the variance in GZB or GZK expression in older adults, we estimated the correlation with participant factors such as age, sex, frailty, CMV serostatus, and plasma TNF or IL-6 using linear regression. The trends observed for both frequency (Figure 3A) and MFI (Figure 3B) were very similar, and only CMV serostatus was positively associated with GZB and GZK expression. With the exception of GZK+ Vδ1+ T-cells, which were significantly less frequent in CMV-positive participants, both GZB and GZK expression tended to be higher with CMV positivity. Specifically, GZB+ cell frequency and MFI was significantly higher for NK-cells and CD4^+^, CD8^+^, and Vδ1+ T-cells, while GZK was significantly higher only in CD8^+^ cells. The associations for CD4^+^ and CD8^+^ T-cell GZB expression were particularly strong, both being >1-SD higher in CMV-positive individuals after adjusting for site of enrollment. Although the sample of young adults was relatively small, the MFI of GZB for CD4^+^ and CD8^+^ T-cells was also significantly higher in CMV-positive participants (standardized β [95% CI]: CD4 = 1.27 [.002, 2.534], CD8 = 1.38 [.42, 2.33]). Finally, since CMV infection and reactivation have been shown to drive perforin expression in NK- and CD8^+^ T-cells, respectively (Pietersma et al., 2010; Ishiyama et al., 2022), and perforin is a critical mediator of the cytolytic response of those cells (McElhaney et al., 2020), we also investigated whether the aforementioned associations between CMV and GZB/GZK+ cell frequency were dependent on perforin expression. There was a tendency for the associations between CMV seropositivity and GZB+ CD8^+^ and Vδ1+ cells or GZK+ CD4^+^ and CD8^+^ cells to be stronger for the perforin positive vs. negative subsets (Supplementary Figure S1A), which was similarly present when considering the ratio of perforin positive to negative cells within each cell population (Supplementary Figure S1B).

### 3.3 Associations between GZB/GZK expression in older adults and influenza antibody titres pre- and post-vaccination

**FIGURE 4:**
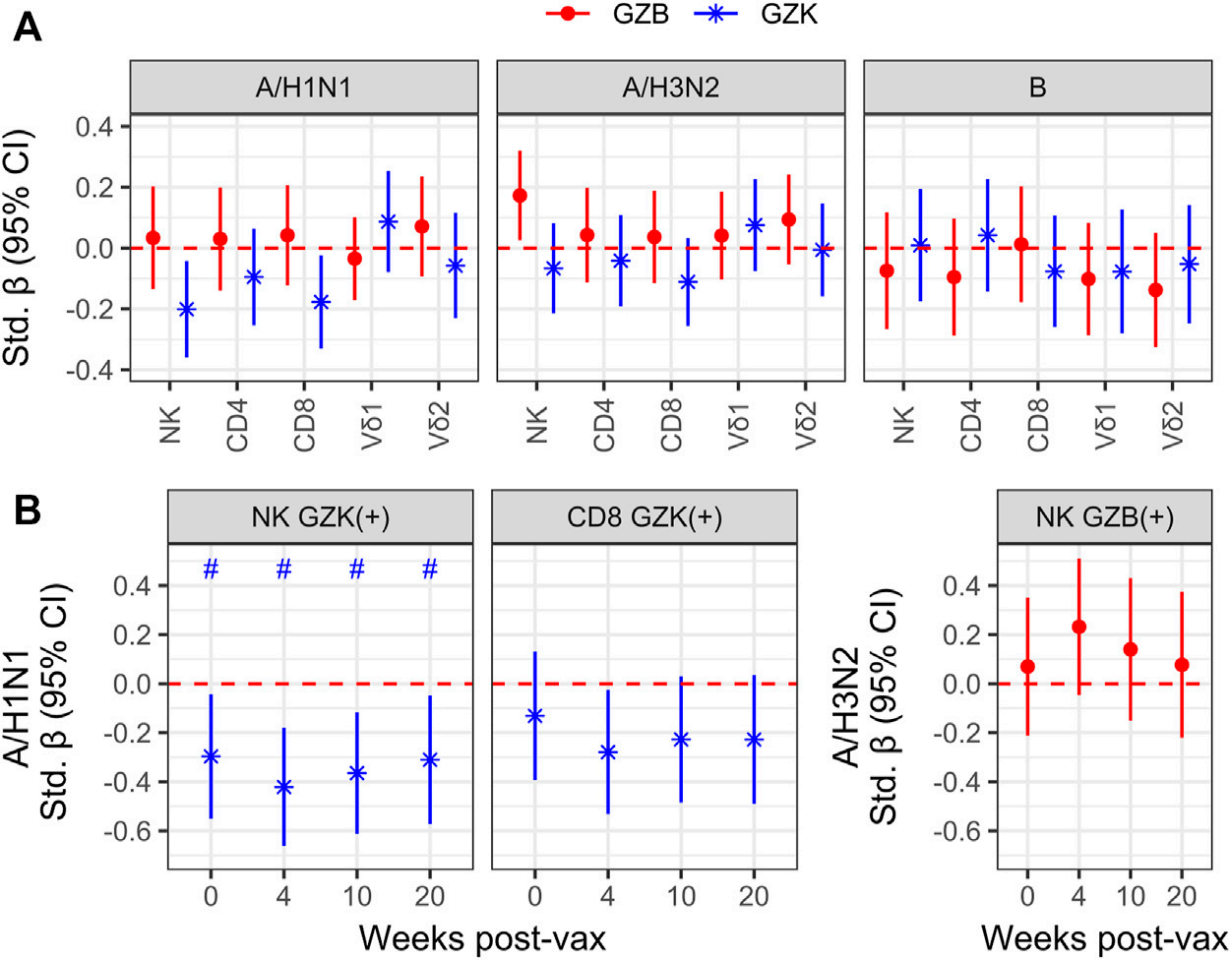
Granzyme-positive cell frequency is associated with vaccine responsiveness and antibody titres in older adults receiving the high-dose vaccine. **(A)** To model vaccine responsiveness, log-transformed titres for influenza A/H1N1, A/H3N2 and B at 4-week post-vaccination were regressed on the log-transformed frequencies of GZB- or GZK-expressing cells, adjusting for age, sex, enrollment site and the log pre-vaccination titres. **(B)** Associations between A/H1N1 titre levels at baseline (i.e., 0), and 4-, 10-, 20-week post-vaccination with the frequency of GZK+ NK-cells or CD8^+^ T cells, omitting adjustment for pre-vaccination titres. No overlap of the 95% CI with the red dotted line indicates significance at the nominal level, while the ‘#’ indicates that significance was maintainted at an FDR adjusted *p* < .05. No significant differences beyond the nominal level were observed in **(A)**.

Although granzymes are most often associated with the cytolytic response to virally infected or transformed host cells, they are also known for their capacity as immunoregulators in a variety of contexts (Omoto et al., 2010; Wensink et al., 2014; Shimizu et al., 2019; Kaiserman et al., 2022). As such, we sought to determine whether the frequency of GZB or GZK+ NK- or T-cells prior to high-dose vaccination was associated with the increase in hemagglutination inhibition (HAI) antibody titres in older adults 4 weeks later. Estimates were mostly non-significant at the nominal level, with three exceptions: the increase in A/H1N1 HAI titres was inversely correlated with the frequency of GZK+ NK-cells (standardized β [95% CI] = −.20 [−.36, −.04]) and CD8^+^ T-cells (−.18 [−.33 −.02]), and the increase in A/H3N2 titres was positively correlated with the frequency of GZB+ NK-cells (.17 [.03, .32]), although none met the FDR-adjusted significance level of *p* < .05 (Figure 4A). Further, these estimates were independent of CMV serostatus, did not appear to differ depending on perforin expression, and only the association of GZB NK-cells with A/H3N2 titres was observed when considering MFI instead of cell frequency (data not shown). Interestingly, when absolute antibody titres at each participant visit (e.g., pre-vaccination, and 4-, 10- and 20-week post-vaccination; Supplementary Table S2) were investigated, the frequencies of GZK+ NK-cells were found to be inversely associated with A/H1N1 titre levels pre- and post-vaccination (standardized β ranging from −.30 to −.42), and maintained significance after controlling the FDR (Figure 4B). The frequency of CD8^+^ T-cells expressing GZK was only associated with A/H1N1 titres 4-week post-vaccination at the nominal level, and NK-cell GZB frequency was not associated with absolute A/H3N2 titres at any time point (Figure 4B).

**FIGURE 5:**
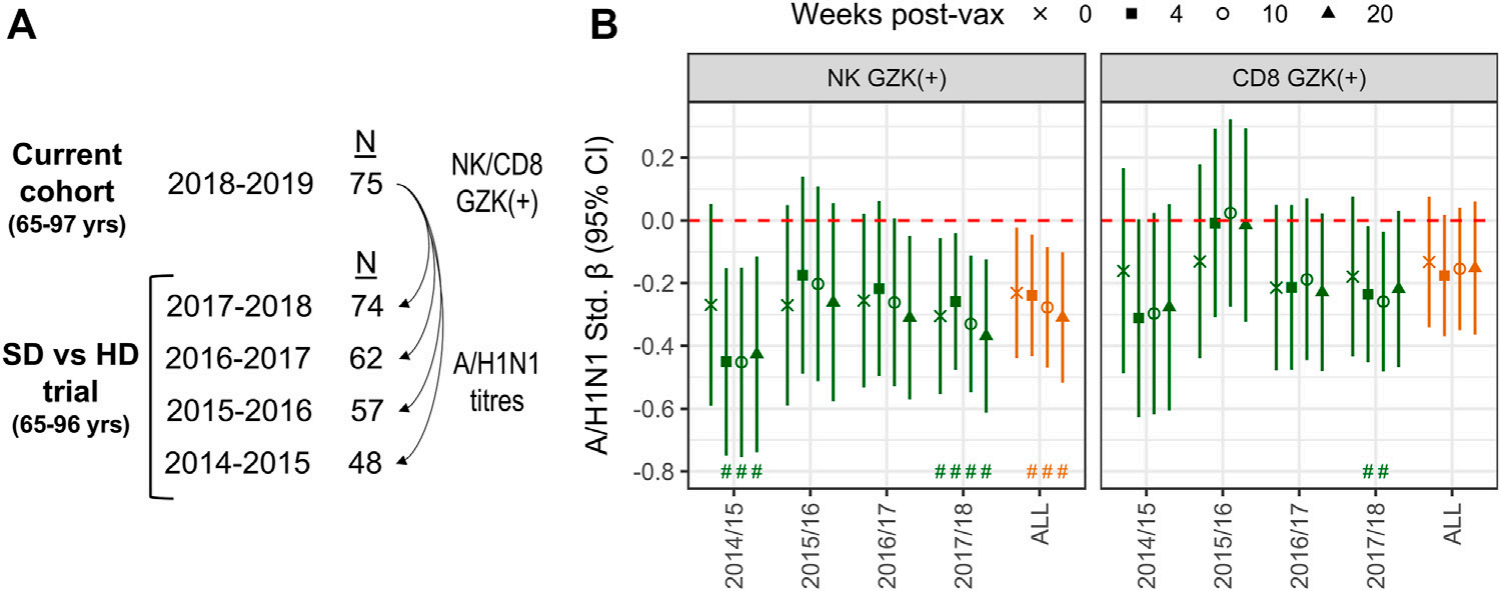
GZK-expressing NK- and CD8 T-cell frequency is inversely associated with prior A/H1N1 antibody titres in older adults. **(A)** Participants enrolled in the current cohort had also been enrolled in multiple years of a previous randomized vaccine trial, in which A/H1N1 antibody titres against the vaccine strain were measured. **(B)** Associations for standardized log A/H1N1 antibody titres measured at baseline (i.e., 0) and 4-, 10-, and 20-week post-vaccination, obtained for each year of the randomized trial or all years combined, with standardized log GZK+ NK-cell or CD8^+^ T-cell frequencies obtained in 2018/19. Models for individual years were adjusted for age, sex, vaccine dose, and site of enrollment, whereas models for all years combined were adjusted for age, sex, dose and random intercepts for site, participant and year of study. No overlap of the 95% CI with the red dotted line indicates significance at the nominal level, while the ‘#’ indicates that significance was maintainted at an FDR adjusted *p* < .05.

The inverse association between GZK+ NK-cell frequency and A/H1N1 antibody titres prior to vaccination is intriguing, as it suggests that this population may reflect the repression of T- or B-cell lineage populations induced by vaccination, or exposure in prior seasons. To explore this hypothesis, we employed data from a randomized trial that compared standard dose and high dose influenza vaccination in the 4 years prior to the current cohort being studied, and in which a number of participants from the current cohort was also enrolled (Figure 5A). Hence, the frequencies of GZK+ NK-cells and CD8^+^ T-cells obtained in 2018 were correlated with retrospective A/H1N1 antibody titres at pre-vaccination and 4-, 10- and 20-week post-vaccination in the years between 2014 and 2018 (Figure 5B; Supplementary Table S2). For GZK+ NK-cells, inverse associations were apparent for most years, many of which maintained significance after controlling for FDR. This was also true when considering all years combined, where standardized coefficients for each time point ranged from −.23 to −.31; importantly, these associations were independent of CMV serostatus and did not appear to differ between standard- and high-dose recipients (data not shown). For GZK+ CD8^+^ T-cells, although the trends clearly suggest an inverse association with antibody titres in previous years, only two time points in 2017/18 were significantly associated, both of which maintained significance after controlling for FDR.

## 4 Discussion

As non-specific proteases, the role of granzymes in health and immunity is complicated and our understanding continues to evolve. Granzyme B is best known as a mediator of cell death, especially triggered by NK-cells and CD8^+^ T-cells, doing so through a variety of mechanisms including the cleavage of caspase proteins and disruption of the mitochondrial membrane (Velotti et al., 2020). While GZB has been shown to be critical in the host response to pathogens such as *Klebsiella pneumoniae*, ectromelia virus and lymphocytic choriomeningitis virus (Müllbacher et al., 1999; Zajac et al., 2003; García-Laorden et al., 2016), it has also been implicated in severe outcomes of SARS-CoV-2 infection (Adam et al., 2021; Kuchroo et al., 2022) and as a mediator of dysfunctional wound healing (Hiroyasu et al., 2021; Turner et al., 2021). Less is known regarding the function of GZK, although it appears to also exhibit cytolytic and immunomodulatory potential (Bouwman et al., 2021) and recently has been described as a key marker of CD8^+^ T-cells and macrophages enriched in inflamed tissues (Turner et al., 2019; Jonsson et al., 2022). Importantly, the expression of both GZB and GZK have been shown to increase with age (Mogilenko et al., 2021; Zöphel et al., 2022), which for GZB, has been postulated as a driver of pathological inflammation (i.e., inflammaging) in older adults (Turner et al., 2021; He et al., 2022). However, little is known of the factors other than chronological aging that lead to increased GZB or GZK expression in older adults, and whether GZB/GZK expression impacts the efficacy of preventative measures against infection such as vaccination.

Building upon prior hypotheses regarding the expression of GZB in CD8^+^ T-cells from older adults and the impact of CMV infection thereon (McElhaney et al., 2012), we found that the frequency of cells expressing GZB and its overall level of expression was significantly higher with age in NK-cells, CD8^+^ and Vδ1+ T-cells, while GZK was higher only in CD8^+^ T-cells. GZB- and GZK-double-positive CD8^+^ T-cells were also significantly more frequent in older adults by > 5-fold. In spite of being limited by a relatively small number of young adult participants, which increases the likelihood of committing a type II error, both GZB (Zöphel et al., 2022) and GZK (Mogilenko et al., 2021) have been previously shown to increase with age in CD8^+^ T-cells. However, it is unclear whether this represents an increase in expression levels across multiple CD8^+^ subsets, or instead an increase in the frequency of particular CD8^+^ subsets that can be characterized by GZB and/or GZK expression. Mogilenko and colleagues (Mogilenko et al., 2021) showed that a subset of CD8^+^ T-cells characterized by the expression of TOX, PD-1 and GZK, but not GZB, are found at higher levels in older mice, regardless of the tissue surveyed. Furthermore, GZK+ CD8^+^ T-cells in human blood were mostly found within the central memory and CD57-negative effector memory compartments, while GZB+ cells were CD57^+^ effector memory with an exhausted TEMRA phenotype (Mogilenko et al., 2021). Jonsson and colleagues (Jonsson et al., 2022) identified a similar CD8^+^ subset characterized by high GZK expression and low GZB expression that were enriched in inflamed tissues, but exhibited low cytotoxic potential. Importantly, in both studies these subsets appear to be distinct from canonical CD8^+^ populations that exhibit abundant levels of GZB when activated.

Previous evidence in bone-marrow chimeric mice suggests that the aged microenvironment, as opposed to cell-intrinsic factors, is likely the primary driving force behind the development of GZK+ CD8^+^ T-cells (Mogilenko et al., 2021). Given this, we sought to characterize how age-related factors in our older adult participants were associated with granzyme expression across NK- and T-cells. Of the factors investigated, which included frailty and circulating levels of the pro-inflammatory mediators TNF and IL-6, only CMV serostatus was significantly associated with GZB or GZK levels. The effect of CMV was substantial though, especially regarding the frequency of NK-cells and CD4^+^, CD8^+^ and Vδ1+ T-cells expressing GZB and CD8^+^ T-cells expressing GZK, which were all significantly higher in CMV-positive participants by .5-1 standard deviations, on average. The prevalence of chronic CMV infection in most countries increases with age (Fowler et al., 2022) and is best known for the inflationary effect it has on the T-cell compartment, particularly in driving clonality in CD8^+^ T-cells, resulting in an increase in subsets exhibiting an exhausted, senescent-like phenotype, while potentially exacerbating a reduction of the overall pool of naïve subsets (Nikolich-Žugich, 2018). Evidence from experimental models (Fehniger et al., 2007) and longitudinal studies (van Leeuwen et al., 2004; Ishiyama et al., 2022; Vaaben et al., 2022) indicates that CMV is an important determinant of the abundance of GZB+ NK-cell and CD8^+^ and γδ T-cells, in line with our own findings. However, ours appears to be the first documentation of the cell-specific patterns of GZK expression in older adults, although a previous study showed that serum levels of GZK increase with CMV infection (Bade et al., 2005). Interestingly, we also found that GZK expression in Vδ1+ T-cells was significantly lower in CMV-positive individuals, which is the opposite of the trends observed for GZB expression in this subset. This suggests that CMV infection may have divergent effects on different granzyme-expressing subsets within the Vδ1 compartment. Although not yet apparent for Vδ1, previous work indicates that there is significant heterogeneity in the Vδ2 T-cell lineage, with subsets identified that primarily express GZK and little GZB, and *vice versa* (Ryan et al., 2016).

Although NK- and CD8^+^ T-cells are most often associated with the cytolytic response to respiratory infection, previous studies suggest that they are also important for vaccine-induced antibody responses to antigens from influenza (Herrero-Fernández et al., 2019; Picard et al., 2022) and other pathogens (Kalia et al., 2021). Given this, we sought to determine whether cell-specific granzyme expression was associated with hemagglutination inhibition (HAI) antibody titres prior to and following seasonal high-dose vaccination in our older adult participants. While not as strong as for CMV serostatus, significant inverse associations between the frequency of GZK+ NK-cells and CD8^+^ T-cells and A/H1N1 antibody responses and positive associations between GZB+ NK-cells and A/H3N2 antibody responses were non-etheless observed. These findings mirrored correlations between the frequencies of cells expressing GZK/GZB and absolute antibody titres post-vaccination, and for GZK+ NK-cells, pre-vaccination as well. This inverse correlation with pre-vaccination titres is particularly intriguing as it suggests that associations between GZK+ NK-cells and vaccine antibody responses in previous years are “echoed” in subsequent collections, which further supports a causal relationship between the two. To test these hypotheses, we performed a retrospective correlation analysis of the frequency of GZK+ NK-cells (obtained in 2018) with A/H1N1 antibody titres from matched participants vaccinated with high-dose or standard-dose formulation in the years 2014–2018. While the strength of association varied year to year, a clear trend was observed where A/H1N1 titres decreased as GZK+ NK-cell frequencies increased. To the best of our knowledge, GZK+ NK-cells as a detriment to vaccine immunogenicity has yet to be reported, although it aligns with what is known regarding the role of NK-cells in germinal centre responses. Thus, in an experimental model of lymphocytic choriomeningitis virus, Rydyznski and colleagues (Rydyznski et al., 2015) found that NK-cell depletion significantly improved the development of cellular and humoral immunity post-infection, which was mechanistically related to the recruitment of follicular T-helper cells to germinal centres and expansion of germinal centre B-cells. Although they did not measure granzyme expression, these effects were perforin-dependent and likely involved NK-cell degranulation, which is in line with the work of Jiang and colleagues (Jiang et al., 2011), who showed that GZK expression increases significantly in NK-cells when they physically interact with activated CD4^+^ or CD8+ T-cells.

Another notable aspect of our findings is the lack of concordance in the direction and nature of the associations related to functionally similar immune features; for example, GZB+ NK-cells were positively associated with A/H3N2 titres post-vaccination, while GZK+ NK-cells were negatively associated with A/H1N1 titres post-vaccination. As we alluded to for CD8^+^ T-cells, GZB and GZK may also represent markers of functionally divergent NK-cell subsets that we cannot unravel at this time. We did observe a significant inverse correlation in the frequency of GZK- and GZB+ NK-cells (data not shown), similarly shown for NKT-cells (Bratke et al., 2005), which would support the notion that they may be markers of separate NK-cell subsets. The reasons underlying differential associations for different NK-cell subsets with A/H1N1 and A/H3N2 antibody responses is less well described in the literature, although it may be related to heterogeneity within the germinal centre B-cell pool following vaccination (Rydyznski et al., 2015). The elicitation of antibodies by vaccination relies mostly on the activation of pre-existing memory B-cell pools, which in older adults are profoundly biased towards group 1 influenza viruses (i.e., A/H1N1 and A/H1N2) as they were the only strains to circulate when those individuals were young (also known as original antigenic sin) (Zhang et al., 2019; Abreu et al., 2020). Hence, if memory B-cells, especially those that are long-lived, are particularly sensitive to the adverse effects of GZK+ NK-cells, inverse associations with antibody titres would likely be more apparent for the A/H1N1 subtype in older adults. Such deduction is merely speculative, but does align with the findings from our retrospective analysis of A/H1N1 antibody titres in prior seasons.

In summary, our work provides new insight into age-related differences in the frequency of GZB- and GZK-expressing lymphoid cell types, and suggests that CMV infection is an important factor driving the expression of both granzymes in older adults, or perhaps distinct cell subsets that are characterized by GZB or GZK expression. We also show that the frequencies of GZK+ NK-cells, and to a lesser extent CD8^+^ T-cells, are inversely associated with vaccine responses using both longitudinal and retrospective analyses. While novel, these findings also represent a limitation of our study as we were unable to determine the mechanistic basis for this relationship or elucidate the phenotype of these cell types beyond their parental lineage; in fact, there may even be a relevant role for the aging- and CMV-related CD56-negative NK-cell subset, which we were unable to identify (Müller-Durovic et al., 2019). An exploratory analysis using immunophenotyping data published as part of a previous study (Picard et al., 2022) showed that the frequency of GZK+ NK-cells most strongly correlated with the frequency of CD56^dim^NKG2A^+^NKG2C^+^ NK-cells (d = .41; data not shown), which suggest a developmentally intermediate (i.e., from CD56^bright^GZK^+^ to CD56^dim^GZK^−^ NK-cells) or even tissue-resident phenotype (Yang et al., 2019; Melsen et al., 2022). Interestingly, we found that two markers of lymph node NK-cells, NKp46 and NKp30 (Eissens et al., 2012; Dogra et al., 2020), were also inversely correlated with A/H1N1 antibody responses in that previous study (Picard et al., 2022). Considering that these markers, as well as NKG2A and NKG2C, are expressed on many other NK-cell subsets, a combined analysis of cellular phenotype and GZK expression would strengthen our current findings, especially if performed in the 4 years of our previously completed randomized vaccine trial. Additional data from longitudinal studies and mechanistic work will be required to validate the findings we have presented and to provide opportunities for intervention to improve responses in older adults.

## Data Availability

The raw data supporting the conclusion of this article will be made available by the authors, without undue reservation.
