# Supplementary material for: NK- and T-cell granzyme B and K expression correlates with age, CMV infection and influenza vaccine-induced antibody titres in older adults
Source: Front Aging. 2023 Jan 5;3:1098200. doi: 10.3389/fragi.2022.1098200 (PMC9849551; doi:10.3389/fragi.2022.1098200)
Supplement: Supplementary file 1 [file Table1.DOCX]

Supplementary Material

# Supplementary Figures and Tables

## Supplementary Figures


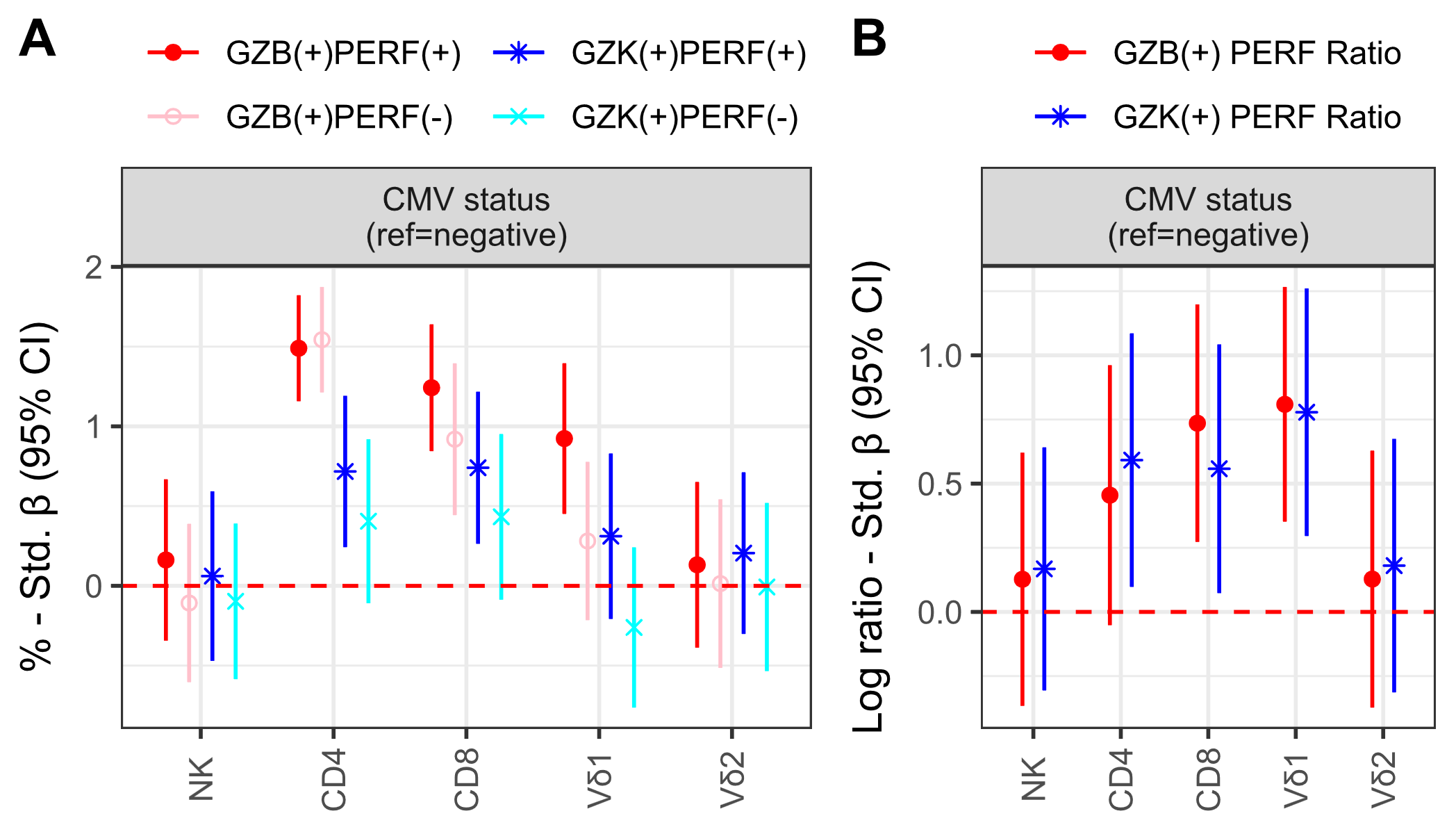


**Supplementary** **Figure 1:** Differential trends in the association between CMV and the frequency of granzyme expressing cells depending on perforin expression. Linear models including CMV serostatus were repeated where A) the standardized log frequency of GZB or GZK-expressing cells, separated according to perforin expression (ie. PERF(+) or PERF(-)), or B) the standardized log ratio of PERF(+) to PERF(-) expressing GZB/GZK cell frequency was the dependant variable. Coefficients and 95% confidence intervals are shown, along with a red, dotted line indicating no significant correlation at the nominal level.

# Supplementary Tables

**Supplementary Table 1:** The geometric mean and standard deviation of cellular granzyme B or K expression, either as the frequency of the cellular population or the mean fluorescence intensity (MFI) of expression.

|  | **Young adults (N=10)** | **Older adults (N=75)** |
| --- | --- | --- |
| **NK [GZB] %** | 83.7 (1.09) | 91 (1.08) |
| Missing | 0 (0%) | 13 (17.3%) |
| **NK [GZK] %** | 3.14 (1.93) | 2.61 (2.1) |
| Missing | 0 (0%) | 12 (16.0%) |
| **NK [GZK(+)GZB(+)] %** | 1.85 (2.06) | 1.89 (2.04) |
| Missing | 0 (0%) | 12 (16.0%) |
| **NK [GZB] MFI** | 53187 (1.42) | 68405 (1.51) |
| Missing | 0 (0%) | 12 (16.0%) |
| **NK [GZK] MFI** | 1240 (1.33) | 1231 (1.36) |
| Missing | 0 (0%) | 12 (16.0%) |
| **Vd1T [GZB] %** | 29.7 (2.25) | 54.9 (1.89) |
| Missing | 0 (0%) | 14 (18.7%) |
| **Vd1T [GZK] %** | 51.4 (1.14) | 51.7 (1.61) |
| Missing | 0 (0%) | 13 (17.3%) |
| **Vd1T [GZK(+)GZB(+)] %** | 19.5 (1.98) | 22.1 (6.38) |
| Missing | 0 (0%) | 12 (16.0%) |
| **Vd2T [GZB] %** | 39.2 (2.07) | 51.1 (1.75) |
| Missing | 0 (0%) | 15 (20.0%) |
| **Vd2T [GZK] %** | 37.7 (1.65) | 38.1 (1.51) |
| Missing | 0 (0%) | 15 (20.0%) |
| **Vd2T [GZK(+)GZB(+)] %** | 13.5 (2.19) | 13.8 (5.79) |
| Missing | 0 (0%) | 12 (16.0%) |
| **Vd1 [GZB] MFI** | 24531 (2.21) | 47382 (2.36) |
| Missing | 0 (0%) | 13 (17.3%) |
| **Vd1 [GZK] MFI** | 9329 (1.43) | 10549 (1.47) |
| Missing | 0 (0%) | 13 (17.3%) |
| **Vd2 [GZB] MFI** | 16103 (2.12) | 23373 (2.1) |
| Missing | 0 (0%) | 14 (18.7%) |
| **Vd2 [GZK] MFI** | 3278 (1.33) | 3776 (1.44) |
| Missing | 0 (0%) | 14 (18.7%) |
| **CD4T [GZB] %** | 0.463 (3.76) | 1.3 (4.98) |
| Missing | 0 (0%) | 12 (16.0%) |
| **CD4T [GZK] %** | 0.277 (2.74) | 0.483 (2.25) |
| Missing | 0 (0%) | 12 (16.0%) |
| **CD4T [GZK(+)GZB(+)] %** | 0.0386 (2.24) | 0.0872 (3.52) |
| Missing | 0 (0%) | 12 (16.0%) |
| **CD8T [GZB] %** | 11.7 (2.14) | 32.6 (2.1) |
| Missing | 0 (0%) | 12 (16.0%) |
| **CD8T [GZK] %** | 3.87 (2.29) | 11 (2.53) |
| Missing | 0 (0%) | 12 (16.0%) |
| **CD8T [GZK(+)GZB(+)] %** | 0.981 (1.84) | 4.98 (2.91) |
| Missing | 0 (0%) | 12 (16.0%) |
| **CD4T [GZB] MFI** | 3232 (1.29) | 5192 (1.84) |
| Missing | 0 (0%) | 12 (16.0%) |
| **CD4T [GZK] MFI** | 856 (1.26) | 934 (1.42) |
| Missing | 0 (0%) | 12 (16.0%) |
| **CD8T [GZB] MFI** | 12489 (2.21) | 32028 (2.36) |
| Missing | 0 (0%) | 12 (16.0%) |
| **CD8T [GZK] MFI** | 1261 (1.22) | 1839 (1.42) |
| Missing | 0 (0%) | 12 (16.0%) |

**Supplementary Table 2:** The geometric mean and 95% confidence interval of hemagglutinin inhabitation antibody titres for all years included in the current study.

|  |  | **Year** | | | | |
| --- | --- | --- | --- | --- | --- | --- |
| **Visit** |  | **2014/15** | **2015/16** | **2016/17** | **2017/18** | **2018/19** |
| **YA (SD)** | | | | | | |
| **A/H1N1** | | | | | | |
| 0 |  | - | - | - | - | 47.6 (24.7, 91.4) |
| 4 |  | - | - | - | - | 102 (46.1, 225) |
| 10 |  | - | - | - | - | 74.6 (37.8, 147) |
| 20 |  | - | - | - | - | 67.3 (35, 129) |
| **A/H3N2** | | | | | | |
| 0 |  | - | - | - | - | 85.7 (29.2, 252) |
| 4 |  | - | - | - | - | 211 (80.1, 557) |
| 10 |  | - | - | - | - | 166 (63, 436) |
| 20 |  | - | - | - | - | 139 (50.2, 386) |
| **B** | | | | | | |
| 0 |  | - | - | - | - | 56.6 (25, 128) |
| 4 |  | - | - | - | - | 126 (66.1, 238) |
| 10 |  | - | - | - | - | 85.7 (43.5, 169) |
| 20 |  | - | - | - | - | 77.3 (37.4, 160) |
| **OA (SD)** | | | | | | |
| **A/H1N1** | | | | | | |
| 0 |  | 52.5 (33.1, 83.2) | 44.9 (29.4, 68.7) | 44 (33.4, 57.9) | 51.1 (37.1, 70.3) | - |
| 4 |  | 104 (65, 166) | 91.9 (57.9, 146) | 94.6 (72.5, 124) | 91.7 (65.2, 129) | - |
| 10 |  | 76.1 (48, 121) | 67.3 (43.5, 104) | 77.5 (58.9, 102) | 71.1 (50.6, 99.9) | - |
| 20 |  | 78 (48.7, 125) | 66.1 (42.2, 104) | 77.5 (58.5, 103) | 72.9 (52.2, 102) | - |
| **A/H3N2** | | | | | | |
| 0 |  | 39 (27.7, 54.9) | 33.2 (22.6, 49) | 42.2 (27.2, 65.4) | 40 (27.5, 58.3) | - |
| 4 |  | 138 (83.4, 228) | 93 (64.2, 135) | 131 (81.9, 210) | 104 (71.9, 149) | - |
| 10 |  | 86.2 (55, 135) | 68.8 (47, 101) | 94.6 (60, 149) | 77.5 (53.1, 113) | - |
| 20 |  | 74.3 (48.8, 113) | 57.2 (38.9, 84.1) | 86.1 (56.8, 131) | 64.8 (43.3, 96.9) | - |
| **B** | | | | | | |
| 0 |  | 29 (22.3, 37.6) | 40.9 (29.6, 56.7) | 42.6 (34, 53.4) | 42.3 (32.5, 55) | - |
| 4 |  | 78 (50.2, 121) | 78.2 (55.2, 111) | 82.6 (63.2, 108) | 70.9 (53.3, 94.4) | - |
| 10 |  | 53.2 (36.3, 77.9) | 65.7 (45.8, 94.4) | 64.8 (50, 84.1) | 60.2 (44.8, 81) | - |
| 20 |  | 54.5 (37.2, 79.8) | 60.1 (43.5, 83) | 55.4 (43.8, 70.1) | 55.6 (40.8, 75.9) | - |
| **OA (HD)** | | | | | | |
| **A/H1N1** | | | | | | |
| 0 |  | 42.9 (28.9, 63.7) | 44.9 (32.9, 61.3) | 37.2 (26.9, 51.5) | 41.4 (27.1, 63.2) | 37.8 (31.9, 44.9) |
| 4 |  | 57.6 (37.1, 89.2) | 82.1 (58.7, 115) | 74.5 (45.2, 123) | 75.5 (49.1, 116) | 85 (69.1, 104) |
| 10 |  | 49.2 (32.8, 73.9) | 66 (48.9, 89.1) | 63 (39.3, 101) | 57.9 (38.8, 86.3) | 62.6 (52.5, 74.7) |
| 20 |  | 48.4 (32, 73.1) | 67.7 (50.3, 91.3) | 58.6 (37.2, 92.4) | 60.1 (38.7, 93.1) | 59.1 (49.9, 69.9) |
| **A/H3N2** | | | | | | |
| 0 |  | 44.4 (27.6, 71.5) | 51 (31, 84.2) | 42.5 (29.5, 61) | 37.3 (24.8, 56.2) | 56 (43.7, 71.9) |
| 4 |  | 85.7 (48.2, 152) | 137 (93.7, 201) | 93.4 (63.1, 138) | 109 (77.2, 155) | 170 (126, 229) |
| 10 |  | 62.6 (35.1, 112) | 90.2 (57.1, 143) | 71 (46.8, 108) | 73.8 (50.4, 108) | 128 (96.4, 170) |
| 20 |  | 66.5 (37.8, 117) | 70 (44.2, 111) | 67.7 (44, 104) | 57.9 (38.8, 86.6) | 108 (82.6, 142) |
| **B** | | | | | | |
| 0 |  | 43.6 (28.7, 66.2) | 53.7 (35.6, 81) | 36.4 (26.8, 49.3) | 38.2 (25.9, 56.3) | 36.1 (30, 43.4) |
| 4 |  | 80 (54.1, 118) | 97 (65.8, 143) | 76.3 (56.1, 104) | 83.8 (58.7, 120) | 66.5 (53.7, 82.3) |
| 10 |  | 60.6 (39.6, 92.9) | 76 (51.7, 112) | 62.2 (46.7, 83) | 64.2 (44.4, 93) | 50.4 (40.9, 62.2) |
| 20 |  | 57.6 (39, 84.9) | 61.5 (43.4, 87.1) | 56.6 (41.4, 77.4) | 60.8 (41.9, 88.1) | 47.1 (38.1, 58.3) |
